# Polo-like kinase 1 (Plk1): an Unexpected Player in DNA Replication

**DOI:** 10.1186/1747-1028-7-3

**Published:** 2012-02-06

**Authors:** Bing Song, X Shawn Liu, Xiaoqi Liu

**Affiliations:** 1 Department of Biological Sciences, Purdue University, West Lafayette, IN 47907, USA; 2 Department of Biochemistry, Purdue University, West Lafayette, IN 47907, USA; 3 Purdue Center for Cancer Research, Purdue University, West Lafayette, IN 47907, USA

**Keywords:** DNA replication, ORC2, phosphorylation, Plk1

## Abstract

Regulation of cell cycle progression is important for the maintenance of genome integrity, and Polo-like kinases (Plks) have been identified as key regulators of this process. It is well established that Polo-like kinase 1 (Plk1) plays critical roles in mitosis but little is known about its functions at other stages of the cell cycle. Here we summarize the functions of Plk1 during DNA replication, focusing on the molecular events related to Origin Recognition Complex (ORC), the complex that is essential for the initiation of DNA replication. Within the context of Plk1 phosphorylation of Orc2, we also emphasize regulation of Orc2 in different organisms. This review is intended to provide some insight into how Plk1 coordinates DNA replication in S phase with chromosome segregation in mitosis, and orchestrates the cell cycle as a whole.

## 1. The cell cycle and DNA replication

The cell cycle plays fundamental roles in many cellular events, such as proliferation, survival and amplification. Deregulation of the cell cycle might lead to abnormal cell growth, which causes cancer or induces cell death through apoptosis. The eukaryotic cell cycle comprises four stages, G1, S, G2 and mitosis. One of the major tasks throughout the cell cycle is to accurately transfer genetic information from parental cells to the next generation. Thus, the two most important stages of the cell cycle are S phase, in which DNA replication occurs, and mitosis, in which the replicated chromosomes are equally segregated into two daughter cells [1].

It is well accepted that DNA replication is initiated bi-directionally at specific loci on chromatin, namely the origins of replication. However, how these origins are selected is still not quite clear. The simplest and best understanding of origins of replication is from study of the budding yeast, *Saccharomyces cerevisiae*. In this organism, replication origins are specified by the autonomous replication sequences (ARS), which are around 100 base pairs and contain a shared 11-base-pair autonomous consensus sequence (ACS). Origin Recognition Complex (ORC) binds directly to ACS to initiate DNA replication. However, even in a simple system such as this, the ACS is not sufficient in itself to predict the origin; the exact location of ACS on the chromosome is also a critical element. Active origins are usually located at intergenic regions, which explains why only 400 out of 12,000 ACS sites are functional in *S. cerevisiae *[2,3]. On the other hand, the origins of replication of the fission yeast *Schizosaccharomyces pombe *differ from those of *S. cerevisiae*. First, the origins are larger in *S. Pombe*, usually from 500 to 1000 base pairs; second, the origins of fission yeast do not have an ARS-like consensus sequence. However, evidence does show that the origins of replication of *S. Pombe *are located mostly at intergenic regions of high A-T content. ORCs with the AT-hook domain preferentially bind to AT-rich islands of DNA to initiate DNA replication [4-7]. The situation is much more complicated in multicellular organisms, as DNA replication can be initiated at any location during early development of *Xenopus laevis *and *Drosophila melanogaster*. But during later development origins of replication are chosen from asymmetric AT-rich regions under the specific influence of epigenetic factors such as gene transcription, nucleosome position, etc. [8-10]. A global search for origins of replication in mouse and Chinese hamster cells revealed that CpG islands with a high GC content but a low methylation level are usually not only the sites for transcription but also for initiation of DNA replication [11,12]. Origins are not all fired at the same time, but instead are activated in early, mid or late stages of S phase. Furthermore, not all origins are activated during S phase. Instead, only a subset is activated during undisturbed replication whereas others, the so-called "dormant origins", are activated in the case of DNA replication stress such as DNA damage [13-15].

DNA replication starts with a series of sequential steps from the formation of pre-replicative complex (pre-RC) by ORC, which recognizes the origin of DNA replication during G1 phase. ORC is a heterohexamer complex contains six members, Orc1 to Orc6. They were originally identified as ARS-binding proteins in *S. cerevisiae *[3], and named sequentially from Orc1 to Orc6 according to their descending molecular weight. ORC homologue proteins (mainly Orc1 to Orc5) were later identified in almost all organisms including *Homo sapiens *[16-18]. All ORCs can bind preferentially to asymmetric AT-rich regions of DNA and function as the loading base for the formation of the pre-RC complex. These proteins are considered to be the only proteins that directly recognize the origins of replication, however in budding yeast Noc3 protein may also be involved [19]. Then cell division cycle 6 (Cdc6) and helicase-loading protein Cdt1 are recruited to the complex, followed by subsequent loading of the minichromosome maintenance protein (MCM) complex. The MCM complex is a heterohexamer that contains six members (Mcm2-7), and has DNA helicase activity, which will later unwind the DNA and allow bi-directional movement of the replication fork. This activation process also involves many other DNA replication factors such as CDC45, GINS complex, SLK2, Treslin, GEMC1, MCM10, DBF4 and RPA. All these proteins ensure proper loading of DNA polymerase and subsequent unwinding of DNA [20]. The formation of pre-RC and activation of the origin of replication have to be tightly regulated to ensure that DNA replicates only once per cell cycle. Once an origin has been activated at a specific locus, it will be inactivated so that re-activation at the same locus will be inhibited within the same cell cycle. During S phase, most Cdc6 proteins are phosphorylated by cyclinA-Cdk2 and exported to the cytoplasm for degradation, whereas a subset of Cdc6 proteins remains bound to chromatin [21]. Cdt1, a critical replication licensing factor, is also regulated by several mechanisms during S phase. Geminin, an inhibitor of Cdt1, binds to and inhibits Cdt1 on chromatin [22,23]. In addition, Cdt1 is ubiquitinated and degraded by SCF^skp2 ^and DDB1^Cul4 ^[24,25]. The MCM complex is phosphorylated by Cdc7-Dbf4 and released from the template when DNA replication is completed [26,27]. From G2 to M phase, Cdc6 and the MCM complex are dephosphorylated. Geminin is ubiquitinated by APC^cdc20 ^so that Cdt1 is released from inhibition [28] and pre-RC can be reformed to prepare for the next round of DNA replication.

## 2. Protein phosphorylation and Regulation of Orc2 in DNA replication

Protein phosphorylation is one of the most well-studied post-translational modifications. It is a reaction catalyzed by a kinase, whereby a phosphate group is added to a serine, threonine or tyrosine residue. The effect on the protein thus modified either enhancement or inhibition of enzymatic activity, or interference with protein-protein interaction, etc., influences various further biological processes.

Since Orc2 is a major component of the DNA replication machinery, its regulation during DNA replication has attracted much investigation. As early as 1999, it was shown that cyclin A-CDK phosphorylates Orc2 in *Xenopus *[29] and that this phosphorylation event inhibits loading of Orc2 onto chromatin. Given the fact that CDKs are the master regulators of the cell cycle, it is not surprising that CDKs regulate DNA replication through phosphorylation of Orc2. Fission yeast Cdc2 phosphorylates Orc2 at four CDK-consensus sites [30]. This phosphorylation starts at S phase and peaks in G2/M to prevent re-replication, thus ensuring that the genome replicates only once per cell cycle. In *S. cerevisiae*, phosphorylation of Orc2 by CDK decreases chromatin loading of Mcm proteins, in contrast, dephosphorylation of Orc2 promotes chromatin loading of Mcm proteins and initiation of DNA replication [31]. The expression level of Orc2 protein is not cell cycle regulated, but studies do show that the localization and association of specific proteins associated with Orc2 change during cell cycle progression. In *S. cerevisiae *, Orc1-6 remain associated with chromatin throughout the cell cycle as a complex [32-35]. However, only Orc2-5 associate with chromatin continuously in mammalian cells. Orc1 dynamically dissociates from chromatin during S phase and re-associates with it at late M/early G1 phase [36]. Mammalian ORCs exist as a variety of complexes including Orc1-6, Orc2-6, or Orc2-5, but the exact role of the ORCs that remain on chromatin after origin firing is still not understood. Studies from yeast and human cells indicate a possible involvement of Orc2 in sister-chromatid cohesion and mitotic chromosome condensation [37,38]. With the concept of both dormant and active origins along replicating DNA, it is also reasonable to speculate that Orc2 stays on chromatin to maintain a dormant origin to counteract DNA replication stress, since it is a critical factor of pre-RC in cases of depletion of other pre-RC components [39,40].

## 3. Plk1 and its role in mitosis

**Figure 1:**
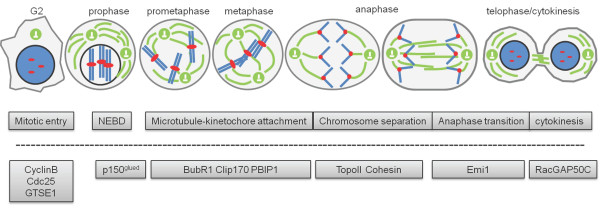
**Functions of Plk1 in mitosis**. Plk1 functions are list below the corresponding mitotic stages. Plk1 substrates and interacting partners are listed below the dashed line corresponding to Plk1 functions. Green: microtubules; blue: chromosomes; red: kinetochores. This listing only focuses on topics covered in this review.

Polo kinase was first discovered in *Drosophila melanogaster *where it had a knock-out phenotype of a mono-spindle pole surrounded by chromosomes to form a circle [41]. The human Polo-like kinase 1 (Plk1) was cloned in 1994, and its expression has been correlated with cell proliferation [42]. Five mammalian Plks have been identified so far: Plk1, Plk2, Plk3, Plk4 and Plk5 [43]. Plk1 and its homologues in budding yeast (Cdc5), fission yeast (Plo1), Drosophila (Polo), nematodes (PLK-1) and Xenopus (Plx1) have been extensively studied [44]. All Plks share a similar domain topology, a kinase domain on the amino-terminal region and a polo-box domain (PBD) on the carboxyl-terminal region. Recent advances with the development of small molecule inhibitors have the advantage of inhibition of Plk1 at post-prophase. The use of these inhibitors has revealed roles of Plk1 in cell cycle regulation, mainly during mitosis, including mitotic entry, centrosome maturation, cohesin release, Golgi fragmentation, microtubule-kinetochore attachment, spindle elongation, and cytokinesis [44,45]. Consistent with these functions, Plk1 localization is also dynamic during cell cycle progression, moving from centrosomes to spindle poles, kinetochores and midbodies [43]. The role of Plk1 during development has not as yet been well studied *in vivo *since knockout of Plk1 in the mouse causes embryonic lethality [46]. During the G2/M transition, Plk1 phosphorylates cyclinB-Cdk1, the key regulator of mitotic entry, and Cdc25, which can dephosphorylate inhibitory phosphorylations on Cdk, consequently resulting in Cdk1 activation for mitotic entry [47]. During nuclear envelop break down (NEBD), Plk1 phosphorylates p150^glued ^, the largest subunit of the dynein/dynactin motor complex, at centrosomes during late G2 phase and promotes movement of p150^glued ^to the nuclear envelop. Plk1 phosphorylation of p150^glued ^facilitates the generation of a deep nuclear envelop pocket or invagination to drive NEBD [48]. During mitotic progression, chromosomes undergo dramatic morphological changes, and chromosome condensation and separation are the critical steps in this process. Plk1 phosphorylation of Topoisomerase II-alpha at Serine 1337 and Serine 1534 increases its decatenation activity, which is critical for sister-chromatid segregation by decatenating the DNA duplexes [49]. Plk1 phosphorylates several kinetochore proteins, such as BubR1, Clip170 and PBIP1 to assist establishment of bipolar spindles during metaphase [50-52]. Replicated sister chromatids are held together by Cohesin. Plk1 phosphorylates one of the Cohesin subunits to promote separation of two sister chromatids [53]. Anaphase-promoting complex/cyclosome (APC/C) is an E3 ubiquitin ligase important for the metaphase to anaphase transition. Plk1 phosphorylation of EMI1, an APC/C inhibitor, leads to its degradation and thus contributes to the activation of APC/C and subsequent metaphase to anaphase transition [54,55]. Plk1 also controls the initiation of cytokinesis through direct interaction with RacGAP50C to regulate the GTPase RhoA [56]. Under conditions of disturbed cell cycle progression, Plk1 has also been reported to regulate DNA-damage checkpoint recovery by targeting G2-and S-phase-expressed 1 (GTSE-1), a p53 negative regulator. This phosphorylation event leads to accumulation of GTSE-1 in the nucleus, where it binds to p53, and subsequently shuttles it out of the nucleus. p53 is degraded in the cytoplasm to inactivate the checkpoint signal and to promote reinitiation of the cell cycle [57] (Figure 1). Topors, which has both ubiquitin and SUMO-1 E3 ligase activity towards p53, is also phosphorylated by Plk1; this enhances its ubiquitination activity and inhibits its sumoylation activity toward p53, resulting in p53 degradation [58].

## 4. Role of Plk1 in DNA replication

Since the major loss-of-function phenotype of Plk1 is cell cycle arrest at mitosis, we know little about the function of Plk1 in DNA replication, another important stage during cell proliferation. Multiple studies have indicated the involvement of polo kinase in DNA replication.

**Figure 2:**
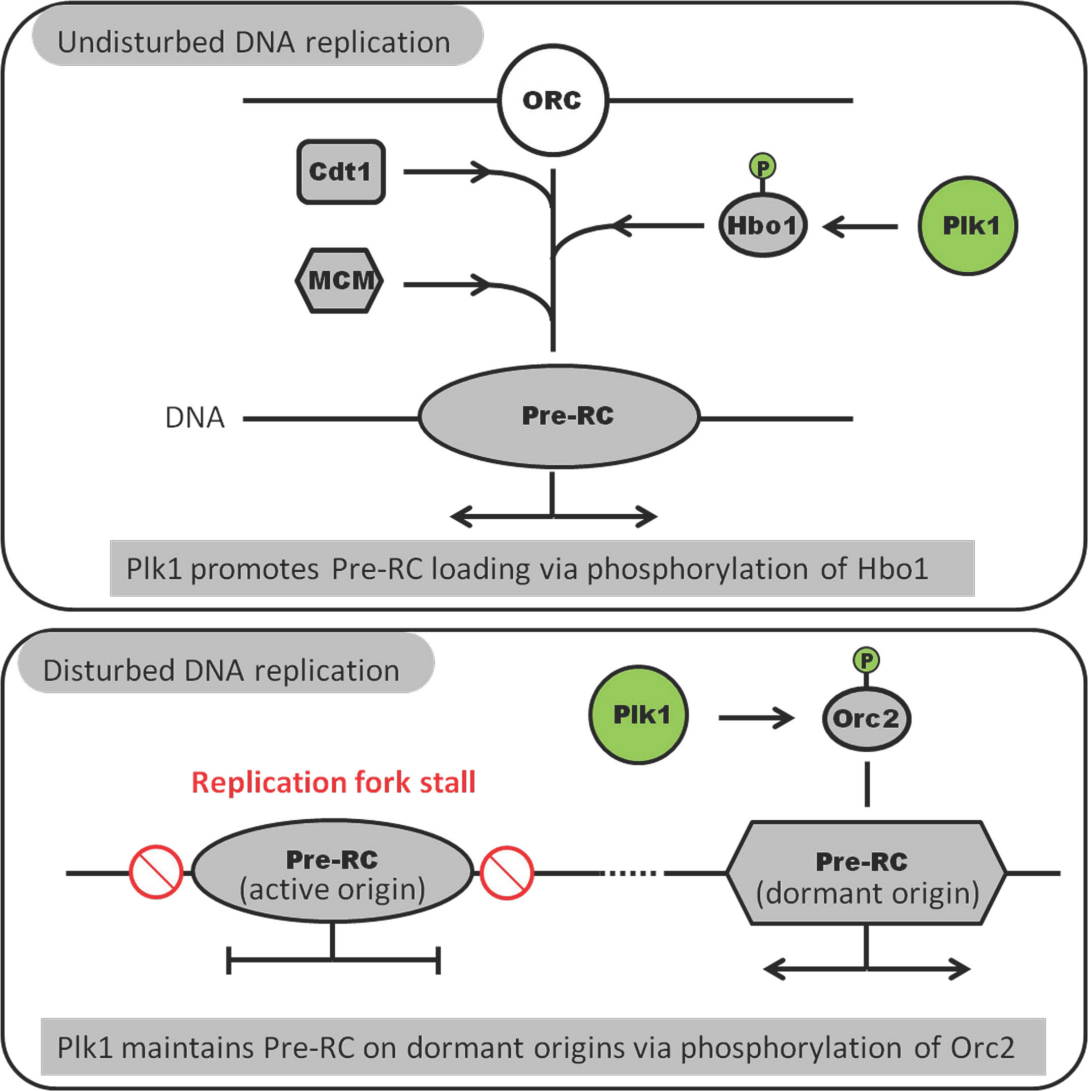
**Functions of Plk1 in DNA replication**. During undisturbed DNA replication, Plk1 phosphorylation of Hbo1 promotes Mcm complex loading thus pre-RC formation. When DNA replication is under stress, checkpoint activation causes stalled replication fork. Plk1 phosphorylation of Orc2 promotes the maintenance of pre-RC on dormant origins.

A possible role of Cdc5, a mammalian Plk1 homologue, at the origin of DNA replication complex in budding yeast was reported as early as 1996 [59]. It was shown that Cdc5 phosphorylates DNA replication initiation protein Dbf4 *in vitro *, suggesting a possible role of Cdc5 in DNA replication. Further studies also showed a synthetic lethal phenotype of Cdc5 with Orc2 but not with other replication factors, such as Dbf4, Cdc7 or Orc5, indicating that Cdc5 and Orc2 might perform related functions during DNA replication in yeast. More interestingly, a Cdc5 mutant has a loss of chromosome phenotype that can be rescued by plasmids containing additional origins of replication. Although mitotic arrest is the most obvious phenotype of Plk1 depletion, long term Plk1 knockdown does lead to slow S-phase progression [60]. Several other publications also reported interactions between Plk1 and replication factors, such as Mcm2, Mcm3, Mcm7 and Orc2, suggesting involvement of Plk1 in DNA replication [61,62]. Yim and Erikson found that depletion of Plk1 with lentivirus induced pre-RC formation defects and reduced DNA replication during subsequent S-phase progression [63]. This result can be partially explained by phosphorylation of histone acetyltransferase binding to the origin recognition complex 1 (Hbo1) by Plk1. Plk1 phosphorylation of Hbo1 at Ser57 regulates pre-RC loading of Mcm2 and Mcm6 proteins [64] (Figure 2). Recent studies have shown that Polo kinase is involved not only in undisturbed DNA replication but also in replication under stress conditions. Aphidicolin treatment leads to stalled replication forks and thus checkpoint activation with activated Chk1. Yoo and colleagues showed that *Xenopus *Plx1 phosphorylates the checkpoint mediator protein Claspin at Ser934 to promote dissociation of Claspin from chromatin and thus inactivation of Chk1. This step ensures that cells recover from replication checkpoint-induced cell cycle arrest. In support of this notion, cells with Claspin S934A mutant do not adapt to the replication checkpoint even after long interphase arrest [65]. It has also been reported that Plx1 can be recruited to chromatin by ATR-dependent phosphorylation of Mcm2 at Ser92. This recruitment is essential for proper DNA replication under stress conditions such as aphidicolin treatment [66]. However, the mechanism of Plx1 as a critical component for DNA replication under stress still needs further investigation. Studies from other organisms also support the role of Plk1 in DNA replication under stress. Avian FANCM protein-deficient cells cannot restart stalled replication forks but instead fire dormant origins to complete DNA replication while the cells are under replication stress. Inhibition of Plk1 by different chemical inhibitors blocks DNA synthesis recovery in FANCM-deficient cells but not FANCM-WT cells [67], suggesting a strong link between Plk1 and dormant origin formation or firing under replication stress. More significantly, we recently found that Plk1 phosphorylates Orc2 at Ser188 and that this phosphorylation event promotes DNA replication under various stress conditions, such as low dose UV, hydroxyurea, aphidicolin and thymidine treatments, in different human cancer cell lines. To understand the mechanism behind this intriguing observation, we demonstrated that Orc2 phosphorylated at Ser188 by Plk1 associates with DNA replication origins and that cells expressing Orc2-S188A fail to maintain functional pre-RC under DNA replication stress. We further showed that the intra-S-phase checkpoint is activated in Orc2-S188A-expressing cells to cause delay of S-phase progression. Our study suggests a novel role of Plk1 in maintenance of genomic integrity by promoting DNA replication under conditions of stress [39] (Figure 2).

## 5. Conclusions

Ever since the discovery of Polo kinase and its knockdown phenotype of cell cycle arrest at mitosis, studies have long been focused on the roles of Polo kinases in different aspects of mitosis. Although Plk1 is activated and peaks at G2/M, its expression starts in early S phase with basal level activity. Increased understanding of the role of Plk1 in DNA replication either under undisturbed or stressful conditions reveals that Plk1 functions not only as a mitotic kinase but also as a coordinator of DNA replication in S phase and mitosis. Thus, Plk1 is a very important propeller of cell cycle progression.

## Competing interests

The authors declare that they have no competing interests.

## Authors' contributions

BS and XSL drafted the manuscript. BS and XL critically edited and approved the manuscript.
